# Supplementary material for: Non-invasive detection of human cardiomyocyte death using methylation patterns of circulating DNA
Source: Nat Commun. 2018 Apr 24;9:1443. doi: 10.1038/s41467-018-03961-y (PMC5915384; doi:10.1038/s41467-018-03961-y)
Supplement: Supplementary file 1 — Supplementary Information [file 41467_2018_3961_MOESM1_ESM.pdf]

Non-invasive detection of human cardiomyocyte death using methylation patterns of circulating DNA

Zemmour et al.

Supplementary Information

Supplementary Figure 1

a

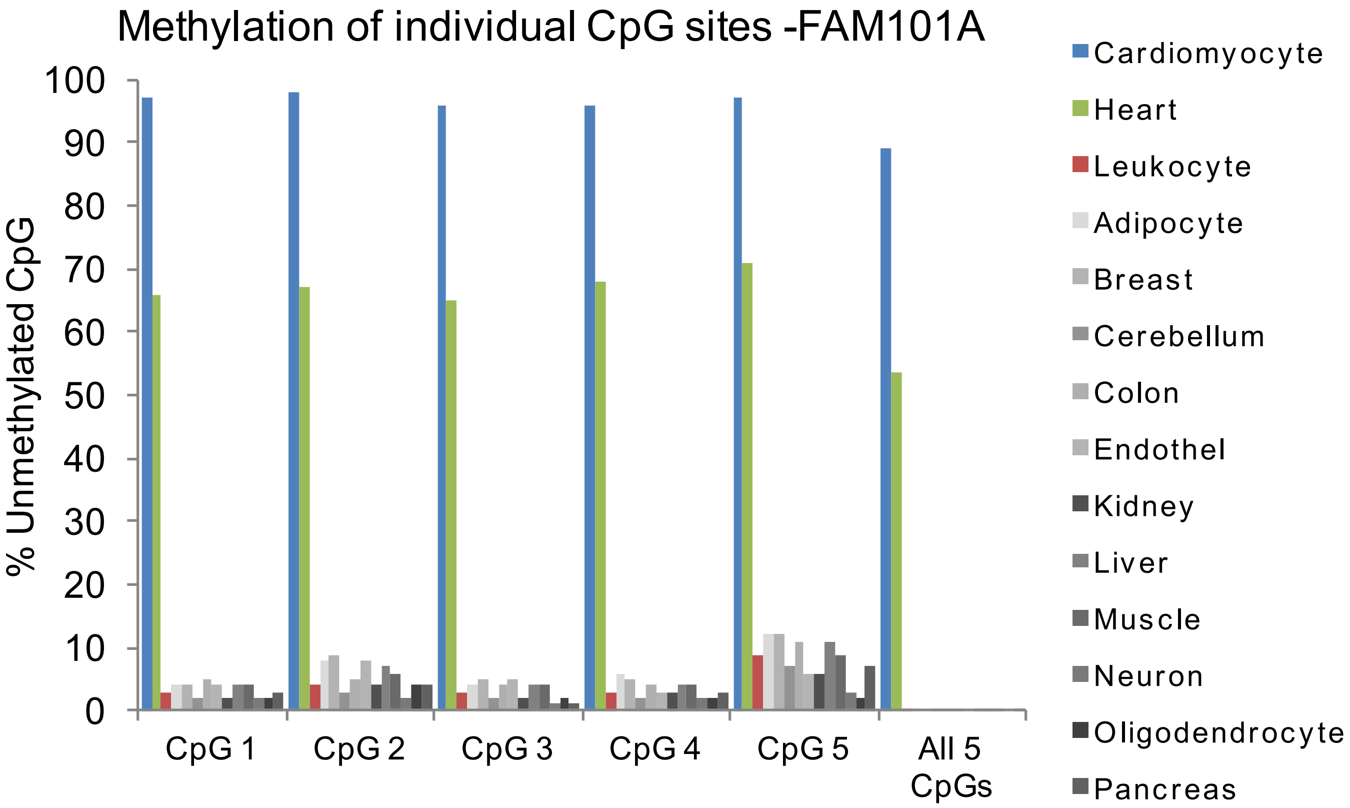

b

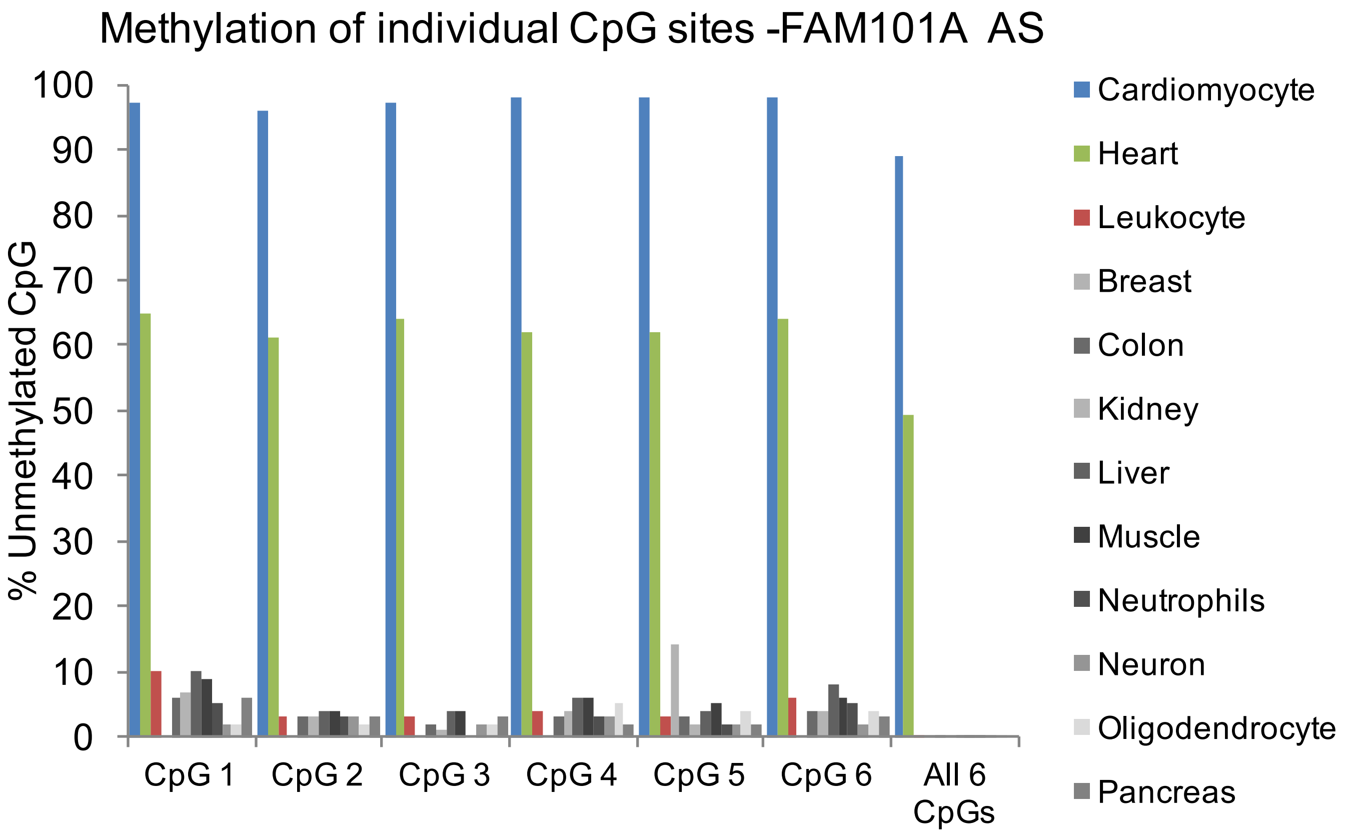

Supplementary Figure 1 (continued)

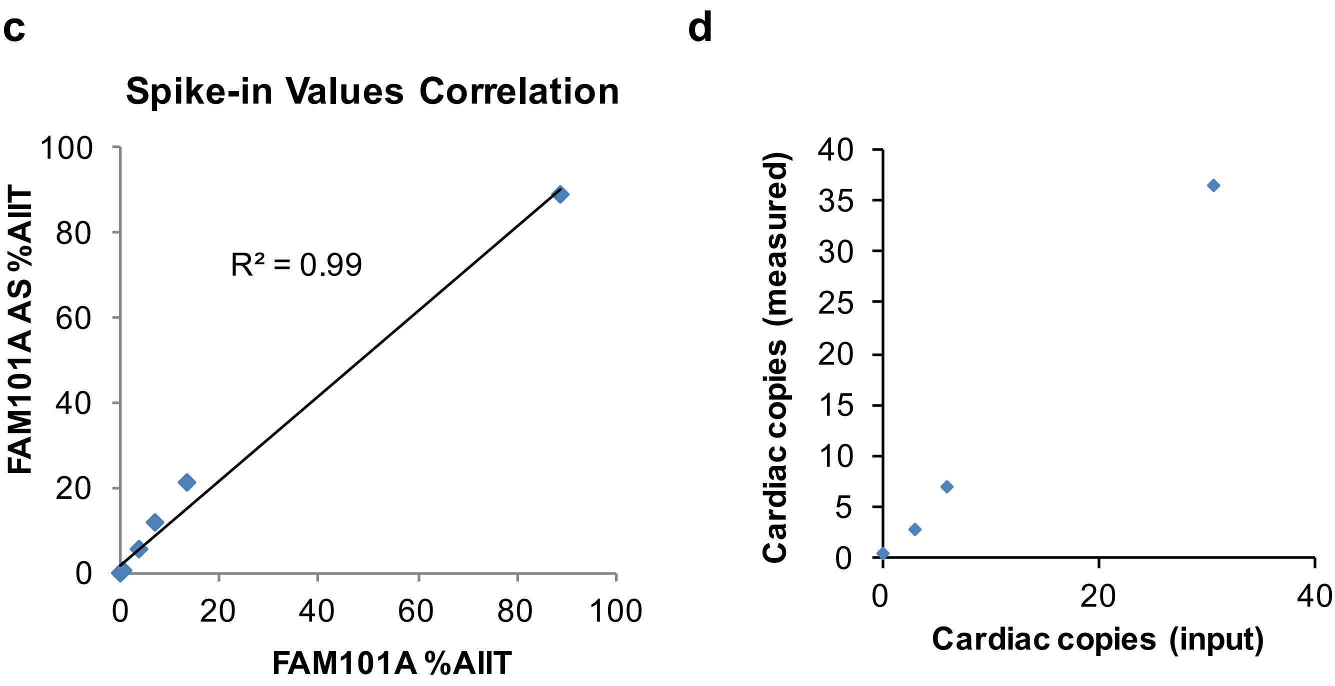

Supplementary Figure 1: methylation of individual and multiple adjacent cytosines within the FAM101A locus.

a. Methylation status of cytosines in the sense strand of FAM101A

b. Methylation status of cytosines in the antisense (AS) strand of FAM101A. Graphs show % of unmethylated molecules in DNA from each tissue. The set of columns on the far right describes the percentage of molecules in which all CpG sites are unmethylated, demonstrating the increase in signal-to-noise ratio afforded by interrogating all CpGs simultaneously.

c. Correlation between results of spike-in experiments using the sense and antisense FAM101A markers.

d. Spike-in experiment measuring the correlation between the number (rather than the fraction) of cardiac genomes in a reaction and the measured signal. The FAM101A antisense marker showed a signal even when only 3 cardiac genomes were present. The signal proved reproducible when there were >10 cardiac genomes in the reaction. It is likely that when fewer copies of the target are present, bisulfite degradation often leads to complete destruction of cardiac DNA such that PCR cannot amplify even a single cardiac molecule.

Supplementary Figure 2

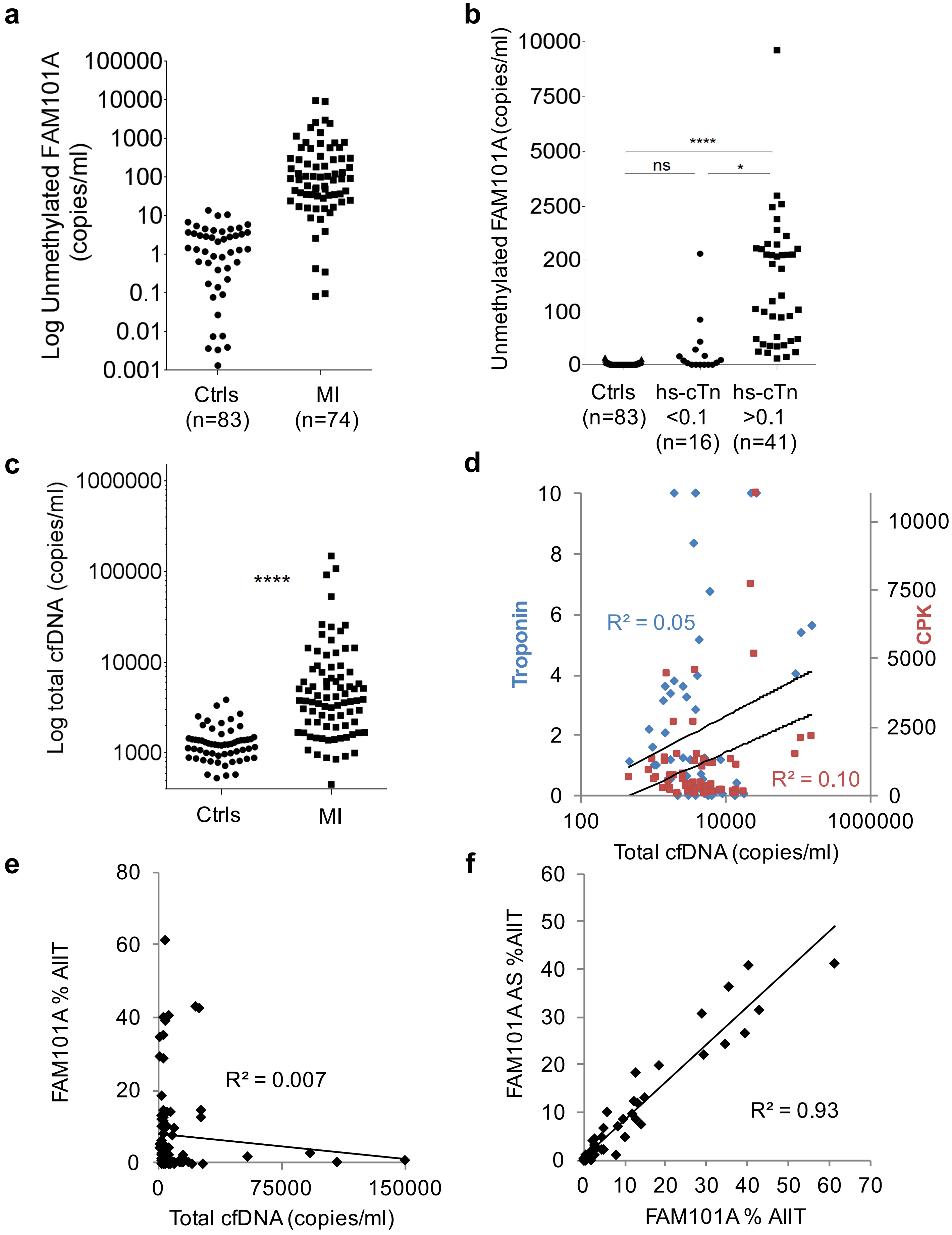

Supplementary Figure 2: additional correlations of cardiac and total cfDNA in STEMI patients.

a. Log scale presentation of unmethylated FAM101A levels in plasma samples from healthy controls (n=83) and patients during STEMI (n=74 samples from 31 patients). 54 values were zero, so are not shown in the graph. Mann–Whitney test for controls vs. patients, *p*-value <0.0001.

b. Cardiac cfDNA levels in controls vs STEMI patients positive or negative for high sensitive troponin using 0.1 as a cutoff. Kruskal-Wallis test *p*-value <0.0001. Dunn's multiple comparisons test: Ctrls vs. Low hs-cTn (<0.1), *p*-value =0.0567; Ctrls vs. High hs-cTn (>0.1), *p*-value <0.0001; Low hs-cTn (<0.1) vs. High hs-cTn (>0.1), *p*-value =0.0001.

c. Total cfDNA concentration in controls and STEMI patients (log scale). Mann–Whitney test for controls vs. patients, *p*-value <0.0001.

d. Lack of correlation between total concentration of cfDNA (genome equivalents/ml) and either hs-Tn (blue) or CK (red) levels. R squared coefficient for logarithmic regression was calculated.

e. Lack of correlation between total cfDNA (genome equivalents/ml) and percentage of cardiac cfDNA. R squared coefficient for logarithmic regression was calculated.

f. Linear correlation between FAM101A sense (S) and antisense (AS) signal in the STEMI samples. R squared coefficient for logarithmic regression was calculated.

Supplementary Figure 3

a

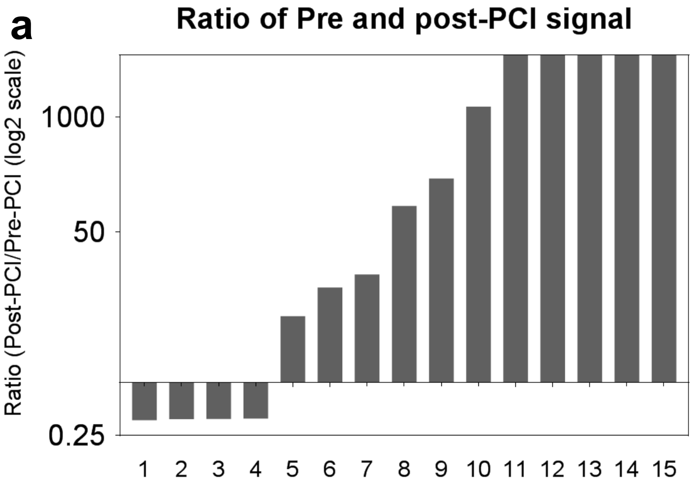

b

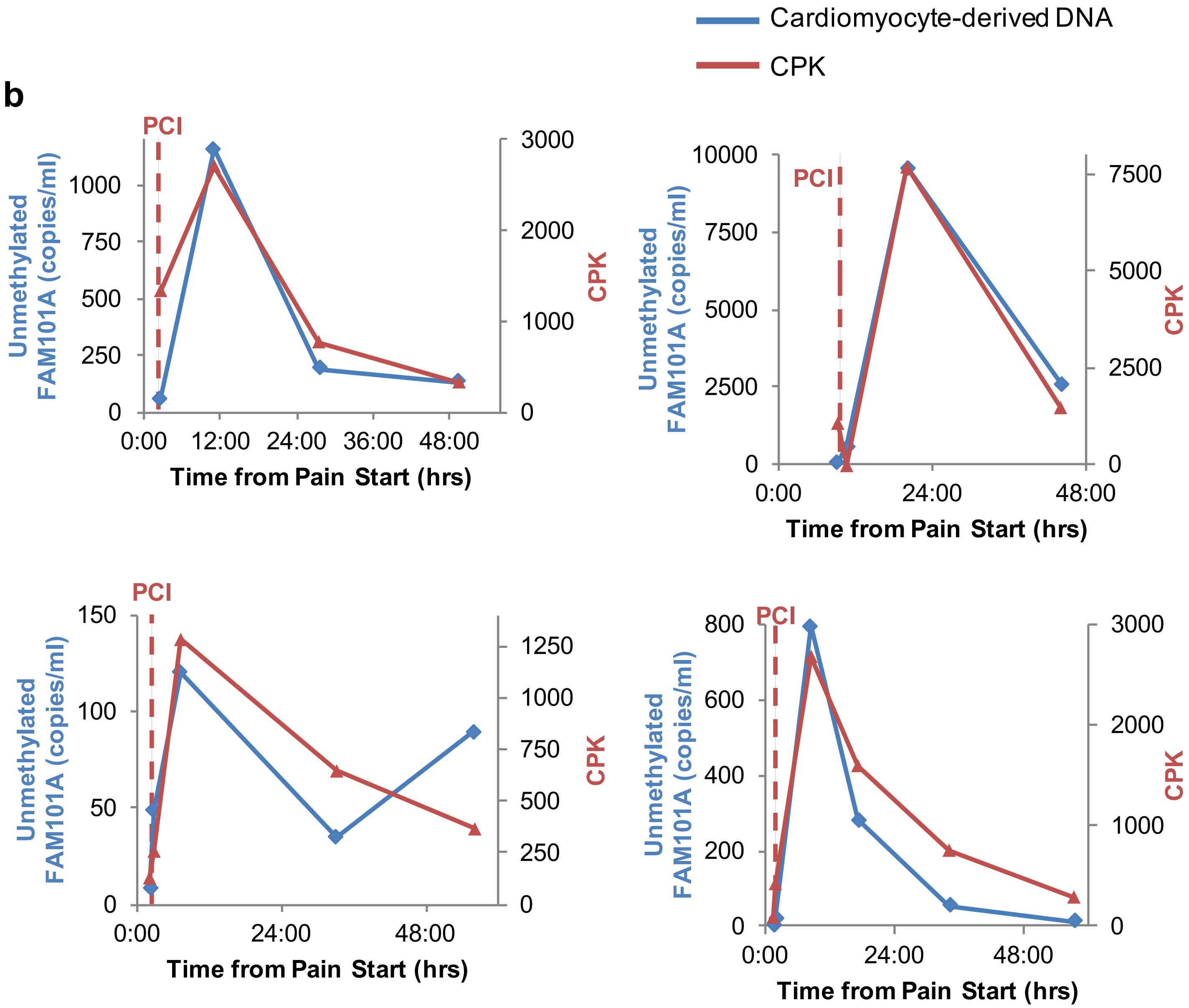

Supplementary Figure 3: Dynamics of cardiac cfDNA and CPK in myocardial infarction.

a. Ratio of cardiac cfDNA before and after PCI in 15 individuals with MI. As expected, cardiac cfDNA levels increased after intervention.

b. Dynamics of cardiac cfDNA and CPK in individual patients. Time 0 is the beginning of chest pain. Vertical dashed line indicates time of PCI.

Supplementary Figure 4

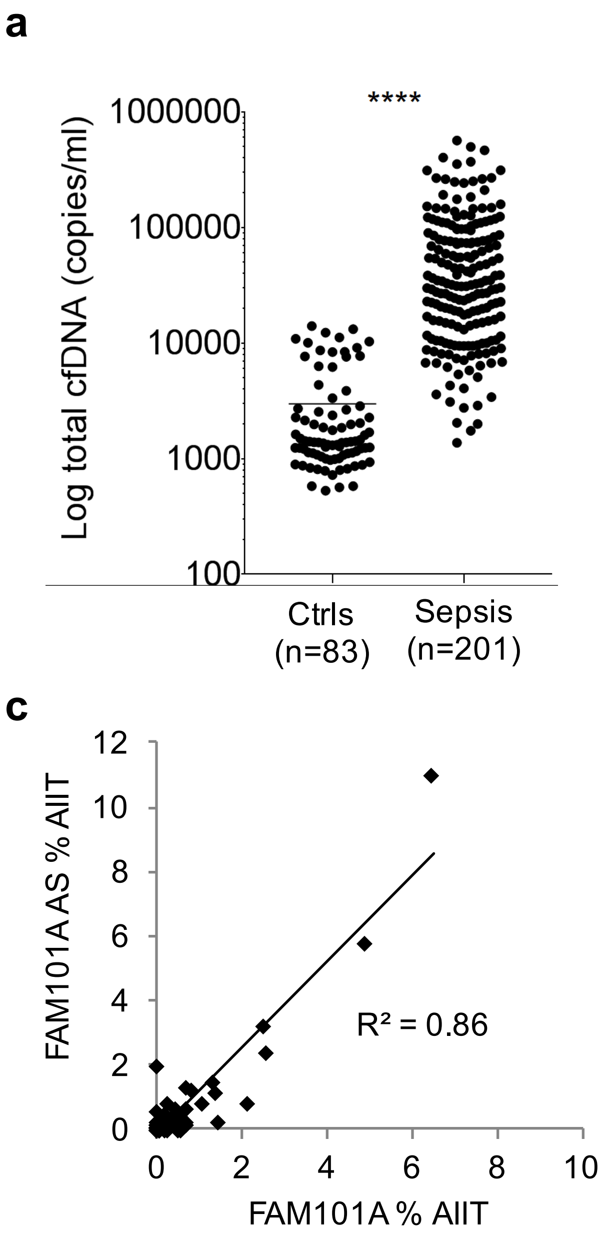

Supplementary Figure S4: Total and cardiac cfDNA levels in patients with sepsis.

a. Total cfDNA concentration in controls and sepsis patients. Mann–Whitney test for controls vs. patients, *p*-value <0.0001. Horizontal line represents the average value of total cfDNA among the samples from healthy individuals.

b. Percentage of cardiac cfDNA in controls and patients with sepsis. Mann–Whitney test for controls vs. patients, *p*-value <0.0001.

c. Correlation between FAM101A sense and antisense signals in sepsis samples. R squared coefficient for linear regression was calculated.

Supplementary Figure 5

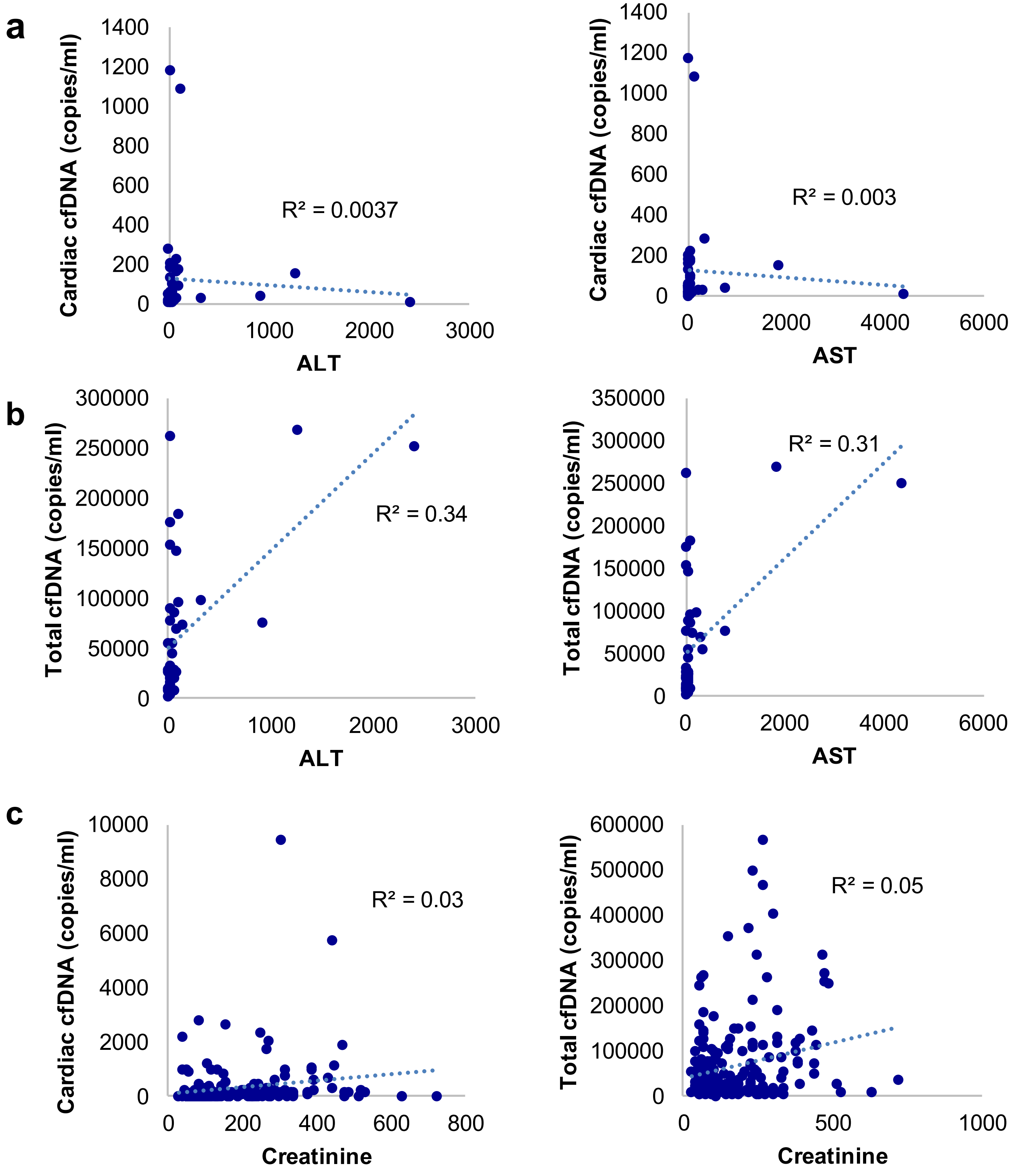

Supplementary Figure 5: Cardiac cfDNA levels do not correlate with the degree of liver or kidney damage in patients with sepsis.

a. Correlation of cardiac cfDNA levels to circulating liver enzymes AST and ALT, which were available for 41 sepsis samples.

b. Correlation of total cfDNA levels to circulating AST and ALT levels.

c. Correlation of cardiac and total cfDNA to creatinine levels (available for 197 samples).

Supplementary Table 1: Exact fraction of unmethylated FAM101A molecules in each tissue

	% Unmethylated molecules	
Sample	FAM101A S	FAM101A AS
Blood	0.01	0.00
Cardiomyocyte	88.96	88.90
Heart	53.72	49.35
Breast	0.00	0.00
Colon	0.20	0.10
Kidney	0.00	0.00
Liver	0.04	0.06
Muscle	0.15	0.10
Neuron	0.01	0.00
Oligodendrocyte	0.00	0.00
Pancreas	0.01	0.00
Vasc. Endothelium	0.00	0.00

Supplementary Table 2: Correlation between cardiac cfDNA levels, total cfDNA levels, troponin and age, and mortality in patients with sepsis.

Cox's proportional hazards multivariate survival analysis, showing that cardiac cfDNA was a strong predictor of mortality, stronger than hs-troponin-T and independent of age. Total cfDNA was not an independent predictor of mortality in that model.

	Wald statist.	P value	Exp(β) (C.I.]
Cardiac cfDNA (copies/ml)	12.3	<0.001	1.00 [1.00 – 1.001]
Total cfDNA (copies/ml)	2.1	0.15	1.00 [1.00 – 1.000]
Age	17.6	<0.001	1.04 [1.02 – 1.06]
Hs-troponin-T	6.4	0.011	2.6 [1.2 – 5.4]
